# Investigating the Effects of Bead Formation on the Physicochemical and Biological Properties of Electrospun Poly(lactic-co-glycolic Acid) (PLGA) Membranes: A Comparative Analysis

**DOI:** 10.1055/s-0045-1804885

**Published:** 2025-05-01

**Authors:** Saaid Al Shehadat, Aghila Rani, Ola Al Shehadat, Ensanya Ali Abou Neel, Sunaina Shetty Yadadi, Natheer Al-Rawi, Khalil Abdelrazik Khalil

**Affiliations:** 1 Department of Restorative Dentistry, College of Dental Medicine, University of Sharjah, Sharjah, United Arab Emirates; 2 Research Institute for Medical and Health Sciences, University of Sharjah, Sharjah, United Arab Emirates; 3 Department of Oral and Craniofacial Health Sciences, University of Sharjah, Sharjah, United Arab Emirates; 4 Department of Mechanical Engineering, College of Engineering, University of Sharjah, Sharjah, United Arab Emirates

**Keywords:** bead formation, electrospun membranes, membrane characterization, physicochemical properties, PLGA scaffolds.

## Abstract

**Objective:**

This study investigates the impact of bead formation on the properties of electrospun poly(lactic-co-glycolic acid) (PLGA) membranes, particularly mechanical strength, uniformity, and cell adhesion, challenging the conventional belief that bead-free membranes are superior.

**Materials and Methods:**

Three types of PLGA membranes were fabricated: beaded (B), fibrous (F), and a mixed (M) configuration of beads and fibers. Morphological, chemical, and surface characteristics were analyzed using scanning electron microscopy (SEM), Fourier-transform infrared spectroscopy (FTIR), and water contact angle measurements. Human dental pulp stem cells (DPSCs) were used to assess *in vitro* cell adhesion, proliferation, and viability across the different membrane types.

**Results:**

SEM imaging revealed distinct morphologies among the different membranes produced via electrospinning. FTIR analysis revealed no significant differences in the chemical composition of the membranes. Contact angle measurements indicated that membranes B and M became more hydrophilic over time, while membrane F remained relatively hydrophobic. All membranes supported DPSCs growth, with membrane M facilitating deeper cell infiltration into the scaffold, indicating enhanced cell integration. Viability assays revealed no significant differences in cell proliferation after 7 days, demonstrating that bead presence did not impair cell growth.

**Conclusion:**

These findings suggest that bead formation in PLGA membranes may offer advantages, such as improved hydrophilicity and enhanced cell infiltration, without compromising cell viability. This study challenges the notion that bead-free membranes are inherently superior and highlights the potential of bead morphology in optimizing electrospun PLGA scaffolds for biomedical applications.

## Introduction

Poly(lactic-co-glycolic acid) (PLGA) is a widely used biodegradable polymer in biomedical applications due to its excellent biocompatibility, tunable degradation rates, and ability to support cell adhesion and proliferation. 1 2 The electrospinning process is a popular technique for fabricating PLGA membranes, allowing for the creation of nanofibrous structures with high surface area-to-volume ratios and interconnected porous networks. These properties are ideal for applications in tissue engineering, drug delivery, and wound healing, where controlled release, cell adhesion, and scaffold degradation are critical. 3 4 PLGA scaffolds offer significant advantages over traditional systems, including biphasic calcium phosphate and bioceramic scaffolds, which have shown potential in enhancing osteogenesis and angiogenesis but may lack the same flexibility in tunable degradation and surface modification. 5 6

The morphology of electrospun fibers, including the presence or absence of beads, can significantly influence the mechanical, physicochemical, and biological properties of the resulting membranes. 7 8 Bead formation during electrospinning is often considered undesirable. Beads can create defects within the fibrous network, potentially reducing mechanical strength, altering pore size distribution, and impacting cellular interactions and material properties. 9 10 11 Consequently, extensive research has focused on optimizing electrospinning parameters to minimize bead formation and produce uniform, bead-free fibers for enhanced membrane performance. 12 13

However, the actual impact of beads on the properties of electrospun PLGA membranes remains under debate. While some studies suggest that bead-free fibers offer superior mechanical and biological performance, 7 11 14 others have found minimal differences in the performance of beaded and bead-free membranes under certain conditions, such as particular mechanical loads, low-stress environments, or nonload-bearing biological applications (e.g., tissue scaffolding or filtration systems). 15 16 Additionally, recent studies have demonstrated that beads can play a functional role in enhancing membrane performance for specific applications, such as water purification through membrane distillation. 17 18 These discrepancies highlight the need for a deeper understanding of how beads influence the overall properties of electrospun membranes, particularly in complex biological environments.

This study aims to systematically investigate the effect of bead morphology on the physicochemical and biological properties of electrospun PLGA membranes. It evaluates the impact of bead formation on hydrophilicity, molecular structure, and surface characteristics, and compares the ability of beaded, fibrous, and mixed membranes to support cell adhesion, proliferation, and viability using human dental pulp stem cells (DPSCs). By challenging the assumption that bead-free fibers are inherently superior, this work highlights the functional benefits of bead morphology and provides new insights into the optimization of electrospun PLGA membranes for biomedical applications, emphasizing their potential for tailored scaffold design.

## Materials and Methods

### Preparation of Synthetic PLGA membranes

To prepare the PLGA membranes, 1 g of PLGA polymer was dissolved in 10 mL of solvent. Two types of solvent systems were used: pure dimethylformamide (DMF) and a mixture of DMF and chloroform in varying ratios (25:75, 50:50, 75:25, DMF). The polymer solutions were stirred continuously overnight to ensure complete dissolution.

Following dissolution, the solutions were electrospun into membranes using an electrospinning machine (Simatic HMI, Siemens, Germany). The process was optimized by adjusting several parameters to achieve the desired membrane morphology. The parameters included the applied voltage (16, 18, 20, 22, and 24 kV), needle gauge (16, 18, 20, and 22), the distance between the nozzle and the collecting plate (10, 12, 14, 16, 18, and 20 cm), and the solution flow rate (0.3, 0.5, 0.8, and 1 mL/h). The drum rotation speed was maintained at a constant 250 rotations per minute throughout the process. Table 1 summarizes the solvent compositions and machine settings used to fabricate three types of PLGA membranes: beaded (B), fibrous (F), and mixed (M).

**Table 1:** Composition and machine settings for synthetic PLGA membranes

PLGA membrane	Solvent 10 mL	Temperature °C	Needle size (gauge)	Humidity	Distance (cm)	Voltage KV	Flow rate mL/h	Drum (RPM)	Time (h/cycle)
B	DMF	35	20	60–70%	18	18	0.3	250	8/2
F	DMF + chloroform (5 mL + 5 mL)	35	18	60–70%	16	22	0.5	250	8/2
M	DMF	45	18	60–70%	18	16	0.5	250	8/2

Abbreviations: DMF, dimethylformamide; PLGA, poly(lactic-co-glycolic acid); RPM, rotations per minute.

After electrospinning, the membranes were allowed to air dry overnight to ensure the complete evaporation of any residual solvents. Subsequently, the membranes were collected and stored at room temperature in a well-sealed box. Handling membrane B, the beaded membrane, posed challenges due to its fragile structure, which made it prone to tearing. To address this, we kept membrane B on the aluminum foil throughout the experiments to maintain its integrity.

### Membrane Morphology and Physicochemical Characterization

#### Scanning Electron Microscopy

Scanning electron microscopy (SEM) was utilized to analyze the surface topography of the three different PLGA membranes. Samples from each membrane type were prepared by cutting them into square pieces, each measuring 0.5 × 0.5 cm. These samples were then coated with a thin layer of gold using a Quorum Technologies SC7620 sputter coater to enhance conductivity. The coated samples were subsequently examined using a SEM (Tescan VEGA 3 XMU, equipped with Oxford Instruments X-max).

#### Fourier-Transform Infrared Spectroscopy

The Fourier-transform infrared spectroscopy (FTIR) analysis was conducted using a Jasco FTIR-6300 (Tokyo, Japan). Samples measuring 1 × 1 cm ^2^ were prepared from the three membranes. To enhance the signal-to-noise ratio, the spectra were collected with a resolution of 2 cm ^−1^ , and 16 scans per sample were obtained in the range of 4000 to 400 cm ^−1^ at room condition at a constant temperature of 25°C, which corresponds to the CH2 group asymmetric and symmetric axial deformations (ν(C-H)). The spectra were collected from three replicates per membrane and data analysis was performed using Origin Pro 8.5 software. The attenuated total reflection sampling mode was employed for spectra analysis, and the obtained spectra were subjected to baseline correction and normalization.

#### Contact Angle Measurement

The water contact angle measurement was conducted to evaluate the wettability of the membranes. Samples measuring 1 × 1 cm ^2^ were prepared from the three membranes and placed on a flat stage. Five microliter droplets of distilled water were introduced onto the surface of the membranes and the sessile drop technique was performed using Rame-Hart automated goniometer (model 290-U1). The mean contact angle value was determined by taking the average of three separate measurements at intervals of 5, 10, 15, 30, and 60 minutes. The experiment was conducted in triplicates for a total number of three samples from each membrane.

### *InVitro* Biological Characterization

#### Cell Culture and Seeding

Human DPSCs (Cat no: 300702) obtained from CLS, Germany, were used in this study. The cells were cultured in Dulbecco's modified Eagle medium/nutrient mixture F12 supplemented with 10% fetal bovine serum and 1% penicillin-streptomycin. The cells were maintained at a temperature of 37°C in a humid atmosphere of 95% O _2_ and 5% CO _2_ . The cells on reaching confluence were subcultured with 1× trypsin-ethylenediaminetetraacetic acid solution (59417 C; Sigma; United States), and the medium was replenished every 2 days.

Before the cell seeding, the three study membranes were prepared in size of 1 × 1 cm ^2^ , sterilized with ultraviolet for 30 minutes, and placed at the bottom of 12-well plates, maintained in position with the help of sterile stainless steel rings. The membranes were incubated in 500 µL of complete culture media for 2 hours in a CO _2_ incubator before cell seeding. The media was then aspirated out and cells were seeded onto the membranes at a density of 1 × 10 ^5^ cells per membrane in a maximum volume of 50 µL and further incubated for 2 hours. An extra 1 mL of the complete culture media was then added and maintained in the incubator for downstream experiments.

#### Cell Attachment to Membranes by SEM

For SEM studies, DPSCs were seeded onto 1 × 1 cm ^2^ size B, F, and M membranes at a density of 5 × 10 ^4^ cells/membrane. The seeded membranes were incubated at 37°C, with 5% CO _2_ and 50% humidity for 3 days. Following incubation, the membranes were gently rinsed with phosphate buffer saline (PBS) and fixed in 2.5% glutaraldehyde (Sigma) for 1 hour at room temperature. After fixation, the membranes were washed with PBS three times and dehydrated through a graded alcohol series. The cell-seeded membranes were coated with gold using Quorum Technologies SC7620 to prepare for SEM imaging as mentioned before.

#### Immunofluorescence Staining Study

For confocal microscopy, cell-seeded membranes for 48 hours were carefully washed three times with PBS and fixed using 4% paraformaldehyde for 20 minutes at room temperature. The membranes were further washed three times in PBS, incubated with FITC-phalloidin (Abcam, United States) for detecting F-actin cytoskeleton for 30 minutes at 4°C. The membranes were then counterstained using 4′,6-diamidino-2-phenylindole (DAPI) for the nucleus (using a mounting medium containing DAPI; Abcam) for 5 minutes. The membranes were then examined under a confocal microscope (Nikon Eclipse Ti-S, Nikon Instruments Inc., United States).

#### Viability Assay of DPSCs on Membranes

For cell viability experiments, DPSCs were cultured at a concentration of 5 × 10 ^4^ cells/membrane on the three different membranes and incubated for 1, 3, and 7 days. Cell viability was assessed using the XTT assay (Roche Diagnostics, Mannheim, Germany). According to the manufacturer's protocol, XTT reagent was added to the cells and incubated for 4 hours. Subsequently, absorbance was measured at 450 nm using a microplate reader (BioTek 800 TS). The mean absorbance values were used to calculate the percentage of cell viability. The experiment was repeated three times.

### Statistical Analysis

The statistical analysis software used in the analysis was SPSS version 24.0 and GraphPad Prism version 8.0. The data collected from the study were expressed as mean ± standard deviation and analyzed using one-way analysis of variance (ANOVA) for intergroup comparison and post hoc Bonferroni test for pairwise comparison. A *p* -value of < 0.05 was considered statistically significant.

## Result

### Preparation of Synthetic PLGA Membranes

**Fig. 1:**
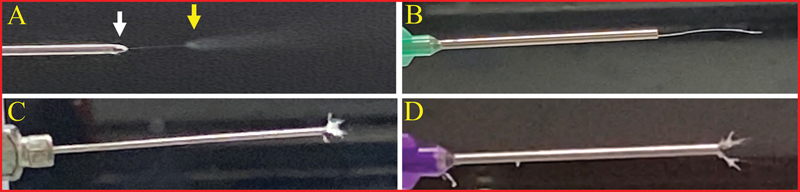
( **A** ) Example of a successful electrospinning process of the synthetic polymer solution with the formation of the Taylor cone (white arrow) and the plume (yellow arrow). ( **B** – **D** ) Examples of unsuccessful processes, identified by the absence of a Taylor cone and/or the accumulation of material buildup at the needle tip.

Only membranes that were produced through a successful electrospinning process were selected for further analysis using SEM. Successful trials were identified based on several key criteria: a smooth and stable electrospinning process, the consistent formation of a Taylor cone at the needle tip ( Fig. 1 ), and the absence of any polymer buildup on the needle or collector. These conditions led to the production of homogeneous membranes, which were free of defects and had uniform morphology suitable for subsequent SEM examination.

### Membrane Morphology and Physicochemical Characterization

#### Scanning Electron Microscope

**Fig. 2:**
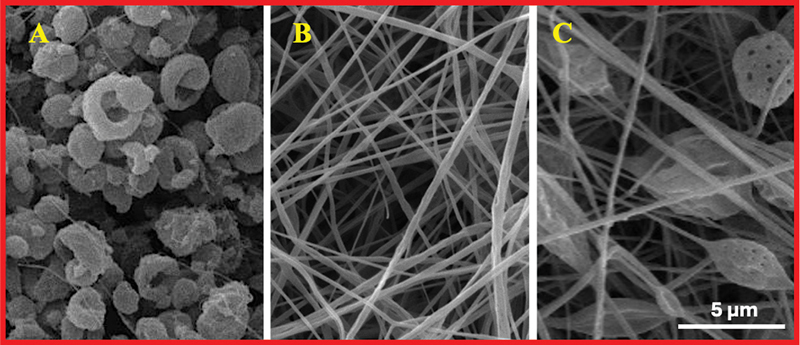
Morphologies of electrospun synthetic poly(lactic-co-glycolic acid) (PLGA) membranes by scanning electron microscopy (SEM). ( **A** ) Beads membrane exhibiting beads. ( **B** ) Fiber membrane. ( **C** ) Mixed (beads and fibers) membrane.

SEM analysis revealed diverse morphologies among the membranes produced via electrospinning. Three different membrane types were identified based on their structural characteristics: one with prominent beaded structures (labeled B), another with predominantly fibrous textures (labeled F), and a third with a combination of beads and fibers, resulting in a mixed morphology (labeled M) ( Fig. 2 ). These variations in morphology underscore the significant impact of the electrospinning parameters on the structural characteristics of the membranes.

#### Fourier-Transform Infrared Spectroscopy

**Fig. 3:**
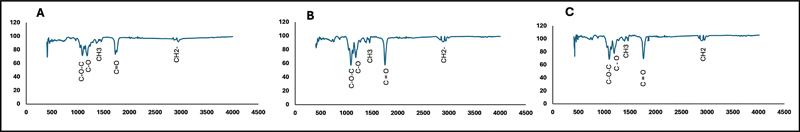
Fourier-transform infrared spectroscopy (FTIR) analysis for the three poly(lactic-co-glycolic acid) (PLGA) membranes. ( **A** ) The bead membrane (membrane B). ( **B** ) The fiber membrane (membrane F). ( **C** ) The mixed membrane (membrane M).

The FTIR spectra of the three membranes ( Fig. 3 ) were analyzed to identify their chemical compositions. All three membranes exhibited characteristic absorption bands, indicative of PLGA's molecular structure. For membrane B ( Fig. 3A ), the spectrum displayed key absorption bands at approximately 1,750 cm ^−1^ , corresponding to C = O stretching (carbonyl groups), 1,180 cm ^−1^ for C-O stretching, and 1,090 cm ^−1^ for C-O-C stretching. Additionally, peaks were observed around 2,945 and 1,380 cm ^−1^ , representing CH _2_ stretching and CH _3_ bending, respectively. Membrane F ( Fig. 3B ) exhibited similar absorption bands to membrane B. The major peaks were observed at approximately 1,750 cm ^−1^ (C = O stretching), 1,180 cm ^−1^ (C-O stretching), and 1,090 cm ^−1^ (C-O-C stretching). The peaks corresponding to the CH _2_ groups at 2,945 cm ^−1^ were also present. For membrane M ( Fig. 3C ), the FTIR spectrum followed a similar pattern to both membrane A and membrane B, displaying key peaks at 1,750 cm ^−1^ (C = O stretching), 1,180 cm ^−1^ (C-O stretching), 1,090 cm ^−1^ (C-O-C stretching), and 2,945 cm ^−1^ (CH _2_ stretching). No significant changes were observed in the chemical composition across the three membranes, suggesting that all three share similar structural characteristics.

#### Contact Angle Measurement

**Fig. 4:**
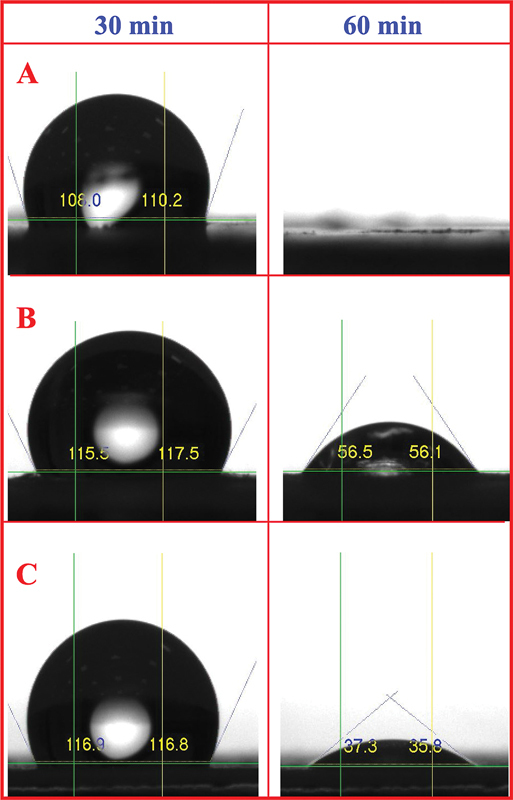
Contact angle measurements for the three poly(lactic-co-glycolic acid) (PLGA) synthetic membranes were taken at 30 minutes (left) and 60 minutes (right). The figure shows the wettability and surface properties of the membranes over time, indicating changes in hydrophobicity or hydrophilicity. Membranes B and M ( **A** and **C** ) exhibited higher hydrophobicity compared to membrane F ( **B** ) at 60 minutes.

The average contact angles of the three membranes are summarized in Table 2 . A significant reduction in contact angle was observed for all membranes after 10 minutes of incubation ( *p* < 0.05), indicating an initial increase in surface hydrophilicity. After 60 minutes of incubation ( Fig. 4 ), the contact angles of membranes B and M showed a further significant decrease compared to membrane F ( *p* < 0.05). These findings suggest that membranes B and M became more hydrophilic over time, while membrane F maintained a relatively higher contact angle, indicating it remained more hydrophobic.

**Table 2:** The average values of water contact angle test and its spread from 0 to 60 minutes

Water contact angle values (m ± SD)			
Time (min)	Membrane		
	B	F	M
0	133.5° ± 0.8°	135.7° ± 2.3°	138.3° ± 0.1°
5	133.7° ± 1.6°	132.3° ± 2.4°	135.3° ± 0.4°
10	129.3° ± 0.5°	129.1° ± 2.0°	132.5° ± 0.4°
15	126.4° ± 0.5°	125.0° ± 1.7°	132.5° ± 1.2°
30	114.7° ± 0.6°	109.1° ± 1.6°	116.8° ± 0.1°
60	35.8° ± 0.8°	105.3° ± 4.6°	36.6° ± 1.1°

Abbreviation: SD, standard deviation.

### Biological Characterization by *In Vitro* Experiments

#### Cell Attachment to Membranes by SEM

**Fig. 5:**
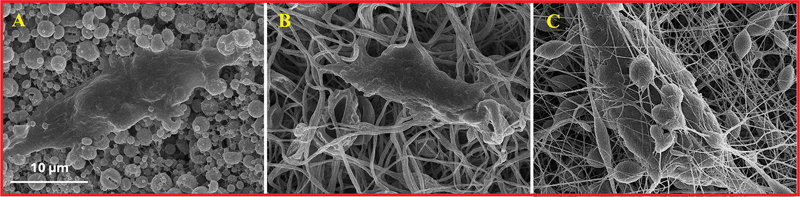
Scanning electron microscopy (SEM) images of dental pulp stem cells (DPSCs) seeded on poly(lactic-co-glycolic acid) (PLGA) membranes after 3 days. ( **A** ) Bead membrane (membrane B), ( **B** ) fiber membrane (membrane F), and ( **C** ) mixed membrane (membrane M).

When DPSCs were seeded onto the membranes, the cells were able to successfully attach and exhibited a flattened morphology, with minimal peripheral blebs and ruffles ( Fig. 5A–C ). Interestingly, SEM images of membrane M revealed that DPSCs not only adhered to the surface but also tended to penetrate the depths of the membrane structure ( Fig. 5C ).

#### Immunofluorescence Staining Study

**Fig. 6:**
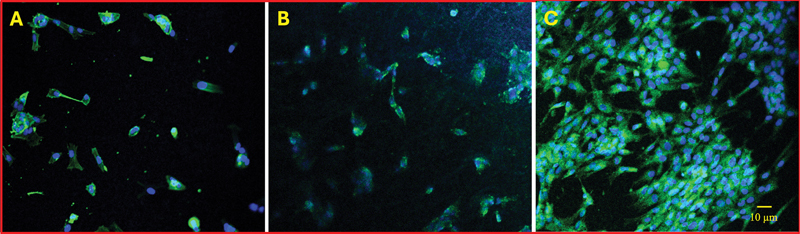
Immunofluorescence staining of dental pulp stem cells (DPSCs) cultured on the synthetic polymer membranes ( **A–C** ) for 48 hours. Cells' nuclei (blue) and mitochondria (green) are shown and demonstrate successful cell adhesion and spreading across all scaffold surfaces. Notably, membrane M (The mixed membrane) exhibited significantly enhanced cell growth and activity, evidenced by a densely populated layer of live cells. ( **A** ) The bead membrane (membrane B). ( **B** ) The fiber membrane (membrane F). ( **C** ) The mixed membrane (membrane M).

Fig. 6 shows the immunofluorescence staining of DPSCs seeded on the three synthetic polymer membranes after 48 hours of incubation. The cells were stained with DAPI (blue) to visualize the nuclei and with FITC-phalloidin (green) to highlight the F-actin cytoskeleton. The confocal microscope images indicate successful cell adhesion and spreading across the surfaces of all three scaffolds. Notably, the actin cytoskeletons in cells grown on membrane M appeared more developed compared to those on the other membranes, as demonstrated by a densely populated layer of live cells ( Fig. 6C ).

#### Viability Assay of DPSCs on Membranes

**Fig. 7:**
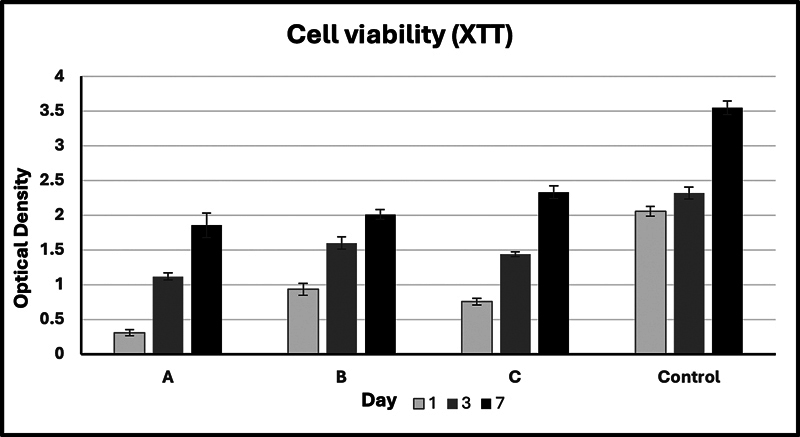
Results of the XTT cell viability assay for dental pulp stem cells (DPSCs) cultured on the different poly(lactic-co-glycolic acid) (PLGA) membranes over 1, 3, and 7 days. A significant increase in cell number was observed from day 1 to day 7 across all groups. The control group showed significant differences compared to all membrane groups at all time intervals. No significant differences between the membrane groups at day 7. ( **A** ) The bead membrane (membrane B). ( **B** ) The fiber membrane (membrane F). ( **C** ) The mixed membrane (membrane M).

Fig. 7 presents the results of the XTT cell viability assay for DPSCs cultured on synthetic PLGA membranes for 7 days. A significant increase in cell number was observed over time from day 1 to day 7 across all groups, including the control. Repeated measures ANOVA between-group analysis revealed a significant mean cell proliferation difference between the groups ( *p* < 0.05). However, post hoc multiple comparisons, with a significance level of 0.05 (two-tailed), indicated no significant differences between the membrane groups at day 7. The control group showed significant differences compared to all membrane groups at all time intervals ( *p* < 0.05).

## Discussion

In recent years, biomaterial scaffolds have garnered significant attention in dental tissue engineering due to their potential to promote tissue regeneration. 19 20 These advancements highlight the importance of ongoing innovation in scaffold design, particularly in optimizing membrane properties to enhance cellular attachment and integration with surrounding tissues. This study aimed to investigate the effects of bead formation on the physicochemical and biological properties of electrospun PLGA membranes. By fabricating three types of membranes, beaded (B), fibrous (F), and mixed (M), we were able to directly compare their structural characteristics, chemical composition, hydrophilicity, and cellular interactions. Based on the literature, the formation of beads could be related to the instability of the liquid jet and altered surface tension and hence incomplete evaporation of the solvent solution before it reached the collector. This led to insufficient polymer entanglement and resulted in the formation of beads rather than fibers. 21 Contrary to the common perception that bead-free fibers are superior in biomedical applications, our findings suggest that the presence of beads may not significantly impair the functionality of electrospun PLGA scaffolds.

The initial SEM analysis of the membranes fabricated under different electrospinning parameters allowed us to select three membranes with distinct morphological characteristics. SEM was utilized to examine the surface morphology of the membranes and to evaluate their structural integrity, providing high-resolution images critical for understanding material properties in tissue engineering applications. 22 During our experiments, membrane B (beaded) presented significant challenges in terms of handling. We were unable to separate membrane B from the aluminum foil used during electrospinning, as it would tear easily during the removal process. This highlights a key limitation in its practical usability, as it lacked the structural integrity required for further manipulation during experimentation. Consequently, membrane B would not be suitable for applications requiring robust handling or mechanical stability. In contrast, both membrane F and membrane M maintained their structural integrity throughout the experiments, with membrane M demonstrating superior handling properties. This makes membrane M a more viable option for practical applications where durability is essential.

The FTIR spectra indicated that all three membranes had similar chemical compositions, displaying characteristic absorption bands associated with PLGA's molecular structure. The spectra showed key absorption bands around 1,750 cm ^−1^ (C = O stretching), 1,180 cm ^−1^ (C-O stretching), 1,090 cm ^−1^ (C-O-C stretching), 2,945 cm ^−1^ (CH _2_ stretching), and 1,380 cm ^−1^ (CH _3_ bending), all of which are typical of PLGA's polymer backbone. 23 24 Contrary to previous studies, 7 10 no significant broad peak around 3,400 cm ^−1^ was observed in any of the membranes, indicating the absence of hydroxyl groups or residual solvents that could suggest surface modification. This confirms that the variations in membrane morphology did not affect the chemical composition or introduce significant surface modifications. Therefore, the differences observed in membrane performance, including hydrophilicity and cellular interactions, are more likely attributed to the physical structure (presence of beads vs. continuous fibers) rather than any substantial changes in chemical composition.

The hydrophilicity of membrane surfaces plays a crucial role in enhancing cell attachment and viability by reducing nonspecific or hydrophobic protein adsorption. 25 In a previous study, the gradual reduction in the PLGA water contact angle over time was described as contact angle relaxation, a phenomenon indicating changes in surface energy or reorientation of surface molecules upon exposure to water. 26 In our study, the contact angle measurements revealed that all membranes were initially hydrophobic, with water contact angles above 90 degrees, 27 consistent with previous reports on PLGA materials. 28 29 However, membranes B and M, which contained beads, showed a significant decrease in contact angle over time, becoming more hydrophilic. This finding suggests that the presence of beads may enhance the exposure of hydrophilic functional groups or alter the surface roughness, facilitating greater water absorption, as supported by Li and Xia. 9 Similar observations have been made with other applications, where bead-containing nanofibers, such as those used in membrane distillation, have demonstrated enhanced surface roughness and water interaction, leading to improved membrane performance. 17

Biological characterization through *in vitro* experiments demonstrated that all three membranes exhibited good biocompatibility, as they effectively supported DPSC attachment and proliferation. SEM images, which are critical for visualizing cell–material interactions, 22 confirmed that DPSCs adhered well to all membrane types and exhibited a flattened morphology with minimal blebbing or ruffling. However, cells on membrane M (mixed morphology, Fig. 5C ) appeared to penetrate deeper into the membrane structure. This finding suggests that the mixed morphology might offer a more conducive environment for cell infiltration, potentially enhancing cell–matrix interactions in ways that purely beaded or fibrous membranes do not, aligning with the findings of Bhattarai et al. 15

The immunofluorescence staining study further revealed that cells on membrane M had more developed actin cytoskeletons and formed a densely populated layer, indicating robust cell spreading and interaction with the scaffold. These results suggest that a mixed bead–fiber morphology could provide a more favorable surface topography for cell adhesion and cytoskeletal organization, potentially improving the scaffold's suitability for tissue engineering applications. 30

The XTT assay was employed to assess cell viability and proliferation on the PLGA membranes. This colorimetric assay is based on the reduction of XTT to a water-soluble formazan product by metabolically active cells, facilitating the quantification of viable cells without the need for additional solubilization steps. 31 The results showed no significant differences in cell viability between the three membrane types at day 7, although all membranes supported substantial cell proliferation over time. Notably, while differences in cell viability were not statistically significant, membrane M (mixed) demonstrated deeper cell infiltration, as evidenced by SEM. This suggests that the mixed morphology may offer an improved microenvironment for cell integration, potentially enhancing scaffold performance in applications requiring high cell–matrix interactions, particularly in long-term applications.

These findings challenge the assumption that bead-free membranes inherently provide better cellular environments, highlighting that membranes with different morphologies can be equally effective under certain conditions. This versatility underscores the potential of electrospun PLGA membranes in biomedical applications, emphasizing that the optimal morphology should be tailored to specific application needs rather than defaulting to bead-free structures.

The results of this study have important implications for the design and fabrication of electrospun PLGA scaffolds. The absence of significant differences in cell viability and the varying impacts on cell morphology and hydrophilicity suggest that bead presence may not negatively impact scaffold performance as much as previously thought. Instead, bead formation could be strategically used to create scaffolds with tailored properties. For instance, the enhanced hydrophilicity and potential for cell penetration observed in beaded and mixed membranes could be beneficial for applications requiring rapid cell infiltration and integration, such as in wound healing or soft tissue regeneration. 14 Future research should explore the long-term effects of bead morphology on scaffold performance, including degradation behavior, mechanical stability, and *in vivo* functionality, to better understand the role of beads in electrospun PLGA membranes.

## Conclusion

This study demonstrated that bead formation in electrospun PLGA membranes does not impair their physicochemical or biological properties and may even enhance hydrophilicity and cell penetration in mixed morphologies. These findings challenge the conventional preference for bead-free fibers and suggest that bead morphology could be strategically leveraged to optimize scaffold performance. Future research should focus on *in vivo* applications and long-term functionality of bead-containing PLGA membranes in biomedical contexts.
